# Pathology confirmation of the efficacy and safety of microwave ablation in papillary thyroid carcinoma

**DOI:** 10.3389/fendo.2022.929651

**Published:** 2022-08-02

**Authors:** Min Ding, Gao-Song Wu, Jian-Hua Gu, Dong-Jie Shen, Rui Zhou, Ying Liu, Rong-Li Xie, Shu-Rong Wang, Hong-Cheng Wang, Jian Fei

**Affiliations:** ^1^ Department of General Surgery, Ruijin Hospital, Shanghai Jiao Tong University School of Medicine, Shanghai, China; ^2^ Department of Thyroid and Breast Surgery, Zhongnan Hospital of Wuhan University, Wuhan, China; ^3^ Department of General Surgery, Shanghai Ruijin Rehabilitation Hospital, Shanghai, China; ^4^ Department of General Surgery, Shanghai Punan Hospital, Shanghai, China; ^5^ Department of General Surgery, Ruijin Hospital Luwan Branch, Shanghai Jiao Tong University School of Medicine, Shanghai, China; ^6^ Department of Medical Ultrasound, Yantai Hospital of Shandong Wendeng Orthopaedics and Traumatology, Yantai, China; ^7^ Department of Medical Ultrasound, Yantai Affiliated Hospital of Binzhou Medical University, Yantai, China; ^8^ Department of Thyroid Surgery, The Second Affiliated Hospital of Fujian University of Traditional Chinese Medicine, Fuzhou, China; ^9^ Department of General Surgery, Pancreatic Disease Center, Ruijin Hospital, Shanghai Jiao Tong University School of Medicine, Shanghai, China; ^10^ Research Institute of Pancreatic Diseases, Shanghai Jiao Tong University School of Medicine, Shanghai, China; ^11^ State Key Laboratory of Oncogenes and Related Genes, Shanghai Cancer Institute, Shanghai Jiao Tong University, Shanghai, China; ^12^ Institute of Translational Medicine, Shanghai Jiao Tong University, Shanghai, China

**Keywords:** thyroid, papillary carcinoma, microwave ablation, surgery, pathology

## Abstract

**Background:**

The incidence of papillary thyroid carcinoma (PTC) has rapidly increased in recent years. Microwave ablation (MWA) was proposed as an alternative treatment for PTC. This study aimed to investigate the efficacy and safety of MWA by exploring the postoperative pathology results of post-ablation lesions in patients with PTC.

**Methods:**

This study retrospectively analyzed data from 12 patients who underwent thyroid surgery after MWA treatment for primary PTC between January 2015 and November 2021 in six hospitals.

**Results:**

The average age of the 12 patients (8 female) was 45.3 ± 9.7 years. There was one patient with PTC (size > 1 cm) and 11 patients with micro-PTC (size ≤ 1 cm), of which eight patients had unifocal micro-PTC and three patients had multifocal micro-PTC. A total of 17 tumor foci with mean size of 6.2 ± 2.6 mm were treated by MWA. The median interval time between MWA and surgery was 6.6 months (range: 0.4–21.9 months). Intraoperatively, adherence to the anterior cervical muscle group was observed in three cases (3/12). Upon postoperative pathologic examination, all the post-ablation lesions of the eight unifocal micro-PTC and two multifocal micro-PTC showed no residual carcinomas. Outside the ablation zone, PTCs were detected in three cases, including two of the eight patients with unifocal micro-PTC and one of the three patients with multifocal micro-PTC. Cervical lymph node metastases were detected in seven patients (7/12).

**Conclusion:**

MWA was feasible for the treatment of primary unifocal low-risk micro-PTC (T1aN0M0) with good efficacy and safety. However, the use of MWA for treating PTC (size > 1 cm) and multifocal micro-PTC remains controversial.

## Introduction

Papillary thyroid carcinoma (PTC) is the most common subtype of thyroid cancer, whose incidence has increased rapidly worldwide in recent years (1). Currently, thyroid surgery is still the first line treatment option for patients with PTC (2). However, most PTCs, especially papillary thyroid microcarcinoma (micro-PTC) with size ≤ 1 cm, often had an indolent clinical course and excellent prognosis, and there were ongoing concerns on whether these cancers were overtreated.

Recently, thermal ablation (TA) was proposed for the treatment of primary PTC, especially low-risk micro-PTC, which has triggered an extensive discussion (3, 4). The low-risk micro-PTC is defined as micro-PTC which showed no lymph node or distant metastasis at diagnosis, no thyroid capsule contact, no extrathyroidal invasion, and no vascular invasion (3). TA, including microwave ablation (MWA), laser ablation (LA), and radiofrequency ablation (RFA), can significantly inactivate local cells through protein denaturation due to the extensive thermal effect. Nowadays, it has been widely used in treating benign thyroid nodules and recurrent micro-PTC with good efficacy and safety (2, 5). The main concerns regarding the treatment of primary PTC are locally incomplete elimination and potential cervical lymph node metastasis, which have been reported in previous literatures (6, 7). Several studies have also reported the efficacy and safety of TA in the treatment of low-risk micro-PTC (8, 9). However, the application of TA for primary PTC remains controversial.

To investigate the efficacy and safety of MWA on primary PTC, we collected data from 12 patients who underwent surgery after MWA treatment for primary PTC and provided insight into the pathological results of post-ablation lesions.

## Materials and methods

### Patients

The inclusion criteria were as follows: (i) patients diagnosed as PTC by ultrasound (US) and fine-needle aspiration biopsy (FNAB) before MWA treatment, (ii) patients who underwent further surgical treatment after MWA, (iii) patients who tolerated surgery, (iv) provided written inform consent for surgery, and (v) those with available postoperative pathological results. We retrospectively analyzed data from 12 patients who underwent thyroid surgery after MWA treatment for primary PTC between January 2015 and November 2021 in six hospitals. The results of thyroid US were recorded according to the risk stratification of the Thyroid Imaging Reporting and Data System (TI-RADS) (10). The outcomes of FNAB were reported according to the 2017 Bethesda System for Reporting Thyroid Cytopathology (11). All the procedures were implemented based on the principle of the Declaration of Helsinki. The requirement to obtain patients’ consent was waived by the relevant institutional ethic committee.

### Procedure of MWA

All procedures were performed by doctors with more than five years of ablation experience. The MWA system used in this study was Nanjing Greatwall MTI-5AT microwave therapeutic instrument with a frequency of 2450MHz ± 30MHz and transmitting power 0-120W which could be adjusted continuously. A 16-gauge cooled-tip needle was used for treatment. The distance between the electrode and the needle tip was 3 mm. Before MWA, contrast-enhanced US (CEUS) examination was performed to evaluate the extent of the tumor and its enhancement mode. High-resolution US was performed to evaluate the relationship between the tumor and critical cervical structures to determine the best puncture site. The patients were placed in a supine position with the neck hyperextended. After skin disinfection, 1% lidocaine was applied for local anesthesia. A mixture of normal saline and lidocaine was injected outside the thyroid capsule according to the nodule’s location to form a ‘liquid isolation zone’ to protect important cervical structures from heat damage. With the guidance of US, a microwave antenna was inserted into the tumor. The output power was 30-40W. The moving-shot or fixed-applicator technique was used for the procedure. The ablated area should exceed the tumor edge until tumors were completely covered by a hyperechoic area. After MWA, CEUS was performed to evaluate the effect of MWA.

### Surgery

The extent of surgery was dependent on the location of the primary PTC before the MWA treatment, preoperative thyroid US and FNA results, and intraoperative freezing pathology results. Lobectomy was performed in patients with unilateral PTC, while total thyroidectomy or near-total thyroidectomy was performed in patients with bilateral PTC, isthmic PTC, and unilateral PTC accompanied with contralateral newly diagnosed carcinomas. All patients received routine ipsilateral central compartment neck dissection. Lateral neck compartment neck dissection was performed only in patients with biopsy-proven metastasis to the lateral cervical lymph nodes (levels II–V).

### Statistical analysis

Statistical analysis was performed using IBM SPSS Version 23.0. Data were expressed as mean ± standard deviation for continuous variables and frequency (percentage) for categorical variables.

## Results

### Baseline clinical characteristics of patients

In this retrospective study, 12 patients (8 females) with an average age of 45.3 ± 9.7 years (range: 33–60 years) were enrolled. Among the 12 patients, one patient (case 11) had multifocal PTC, with mean size > 1 cm, and 11 patients (case 1–10, 12) had micro-PTC (size ≤ 1 cm), of which eight patients (case 2, 4–10) had unifocal micro-PTC and three patients (case 1, 3, 12) had multifocal micro-PTC. A total of 17 tumor foci with a mean size of 6.2 ± 2.6 mm (range 3–11 mm) were treated by MWA. All cases showed no evidence of cervical lymph node or distant metastasis, thyroid capsule contact, extrathyroidal invasion or vascular invasion based on US and FNAB evaluation before MWA treatment. The clinical tumor-node-metastasis stage (cTNM) of all patients was T1aN0M0 prior to MWA except for case 11 (T1bN0M0). The median interval time between MWA and surgery was 6.6 months (range: 0.4–21.9 months) **(** **Table 1** **).**

**Table 1:** Baseline clinical characteristics of enrolled patients.

case	Sex	Age (years)	pre-ablation US			preoperative US			Interval days (months)
			location	size(mm)	TI-RADS	size(mm)	TI-RADS	suspicious LNM	
1	female	46	left, upper pole	5.0×4.0	4A	7.0*6.0	4B	No	6.77
			right, upper pole	7.0×9.0	4A	15.5*7.3	4B		
2	female	44	right, upper pole	4.2×3.9	4A	11.3*3.2*5.5	4A	No	3.47
3	female	58	left, middle pole	4.2×4.1×4.5	4A	18.2*5.5	/	No	16.63
			right, middle pole	5.3×5.7×4.8	4A	3.4*3.0	/		
4	female	37	right, lower pole	3.0×1.5	4A	2.3*3.2*2.0	/	Yes	10.90
5	female	35	left, near to isthmus	5.1×3.3×4.1	4B	20.0*13.0	/	No	1.33
6	female	57	left, lower pole	9×8.8×7.7	4C	15.0*13.0*14.0	4C	No	6.40
7	male	53	left, middle pole	5.3×4.2	3	7.0*6.0	4A	Yes	9.70
8	male	44	left, middle pole	6.0×4.0	4B	8.0*8.7*8.0	4A	Yes	21.93
9	female	34	isthmus	5.4×6.3×5.9	4C	11.7*4.3*11.8	/	Yes	5.10
10	male	60	right, middle pole	5.9×5.0×8.1	4B	18.7*16.9*11.6	/	No	2.27
11	male	33	left, middle pole	11×7.0	4A	4.9*4.7*2.7	/	Yes	15.27
			right, lower pole	11×6.0	4A	3.0*1.5	/		
12	female	43	left, lower pole	3.0×2.0×3.0	4A	11.0*9.0*6.0	/	No	0.43
			right-1, middle pole	2.4×3.5	4B	9.0*14*5.0	/		
			right-2, middle pole	6.2×5.3×0.49	4A	12.0*15.0*11	/		

US, Ultrasound; TI-RADS, Thyroid Imaging Reporting and Data System; LNM, lymph node metastasis.

* means multiply.

### Reasons for surgical treatment

The reasons for further surgical treatment could be divided into five categories: (i) new thyroid carcinoma diagnosed by FNA (case 4) or suspected recurrence in the post-ablation zone detected by US (case 1 and 6); (ii) clinically evident or suspected cervical lymph node metastases during follow-up (5/12, case 4, 7-9, 11), among which three cases (case 4, 7, 8) were diagnosed as lateral cervical lymph node metastases; (iii) patients were worried about the efficacy of MWA and preferred to undergo further surgery for the definite diagnosis of post-ablation lesions (3/12, case 2, 5, 10); (iv) Case 3 developed nodular goiter, which increased in size after MWA and resulted in local compression; (v) MWA was an alternative treatment for patients who could not tolerate surgery after evaluation, and one patient (case 12) preferred to undergo further surgical treatment after her general condition improved.

### Intraoperative findings and postoperative pathology results

**Figure 1:**
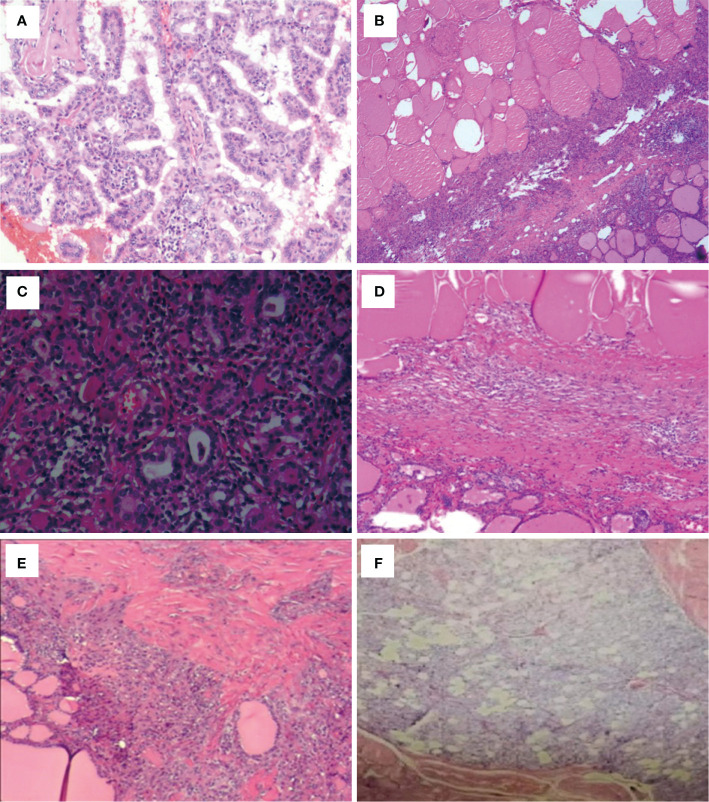
Post-ablation lesions of unifocal micro-PTC showed no residual carcinomas according to the postoperative pathology results (case 4–9). **(A)** Chronic inflammation in case 4. **(B)** Chronic inflammation and fibrosis in case 5. **(C)** Hashimoto’s thyroiditis and fibrosis in case 6. **(D)** Coagulative necrosis and fibrosis in case 7. **(E)** Fibrosis and nodular goiter in case 8. **(F)** Fibrosis and granuloma in case 9.

**Figure 2:**
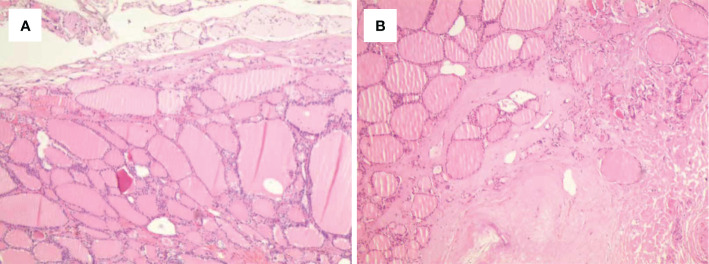
Histological outcomes of the post-ablation lesions in multifocal micro-PTC (case 3). **(A, B)** Nodular goiter was found in the post-ablation zone of the left **(A)** and right **(B)** thyroid lobes.

**Figure 3:**
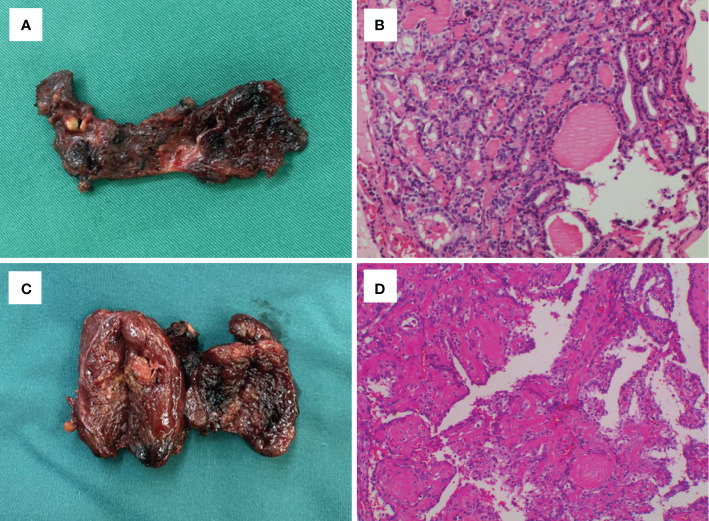
Gross findings and histological outcomes in case 11 **(A, B)** and case 12 **(C, D)**, which showed tumor recurrence in the post-ablation lesions.

None of the cases developed hoarseness after MWA treatment or showed recurrent laryngeal nerves involvement during surgery. Post-ablation lesions were found to adhere to the anterior cervical muscle group in three cases intraoperatively (3/12; case 5, 6, 9). Four patients underwent total thyroidectomy (case 1, 4, 11, 12), and three patients underwent near-total thyroidectomy (case 3, 5, 9). The remaining patients (5/12) underwent lobe thyroidectomy. All unifocal micro-PTC post-ablation lesions (8/12; case 2, 4–10), including case 6, showed no residual carcinomas according to the postoperative pathology results (**Figure 1**). In addition, two multifocal micro-PTCs (case 1, 3) were also disease free in the post-ablation area (**Figure 2**). Residual tumors were detected in two cases (2/12; case 11, 12) (**Figure 3**). Outside the ablation zone, new PTCs were detected in three cases, two (case 4, 7) of the eight patients with unifocal micro-PTC and one (case 12) of the three patients with multifocal micro-PTC. Seven cases (7/12; case 1, 4, 7–9, 11, 12) developed cervical lymph node metastases, of which lateral cervical lymph node metastases were found in three cases (case 4, 7, 8) (**Table 2**). Therefore, three cases (3/12; case 2, 5, 10) were totally disease free after MWA treatment, but still underwent subsequent surgical treatment.

**Table 2:** Intraoperative findings and postoperative pathology results.

Case	Location	Surgery	Ablation injury	Residual	Pathology	New PTC	LNM	pTNM
1	left	TT	no	no	hyaline degeneration, fibrosis	no	CLNM	T1aN1aM0
	right			no	hyaline degeneration, fibrosis			
2	right	LT	no	no	fibrosis	no	no	T1aN0M0
3	left	near-TT	no	no	fibrosis	no	no	T1aN0M0
	right			no	fibrosis			
4	right	TT	no	no	chronic inflammation	yes	LLNM	T1aN1bM0
5	left (near the isthmus)	near-TT	strap muscle	no	fibrosis	no	no	T1aN0M0
6	left	LT	strap muscle	no	fibrosis	no	no	T1aN0M0
7	left	LT	no	no	coagulative necrosis, fibrosis	yes	LLNM	T1aN1bM0
8	left	LT	no	no	necrosis, fibrosis	no	LLNM	T1aN1bM0
9	isthmus	near-TT	strap muscle	no	fibrosis, granuloma	no	CLNM	T1aN1aM0
10	right	LT	no	no	necrosis, fibrosis	no	no	T1aN0M0
11	left	TT	no	yes	micro-PTC	no	CLNM	T1bN1aM0
	right			no	fibrosis			
12	left	TT	no	yes	micro-PTC	yes	CLNM	T1aN1aM0
	right 1			yes	micro-PTC			
	right 2			yes	micro-PTC			

TT, total thyroidectomy; LT, lobe thyroidectomy; PTC, papillary thyroid carcinoma; LNM, lymph node metastasis; CLNM, central cervical lymph node metastasis; LLNM, lateral cervical lymph node metastasis; pTNM, pathological tumor-node-metastasis stage.

## Discussion

This multicenter retrospective study pathologically confirmed that MWA could achieve complete local tumor elimination for primary unifocal low-risk micro-PTC (T1aN0M0). However, MWA treatment for PTC (size > 1 cm) and multifocal micro-PTC was still limited. To our knowledge, this study is the first to systematically analyze the postoperative pathology results of patients with PTC who underwent MWA treatment. TA was not recommended for the treatment of primary PTC in the 2015 American Thyroid Association (ATA) management guidelines because there was still insufficient medical evidence (2). Nevertheless, the efficacy and safety of TA on primary low-risk micro-PTC have been intensively studied in recent years, which showed excellent outcomes (5, 12). The 2019 Chinese expert consensus also claimed that a prospective clinical study should be conducted in patients with micro-PTC who meet specific criteria (13). Our study demonstrated the efficacy and safety of MWA on unifocal low-risk micro-PTC pathologically, providing evidence for new guidelines on the management of unifocal micro-PTC.

In our study, complete local tumor elimination was found in all unifocal micro-PTC (8/12). Yue et al. (14) conducted a long-term prospective study involving patients with unifocal low-risk micro-PTC treated with MWA. In this study, three patients underwent surgery, and two patients underwent core needle aspiration (CNB) in the follow-up visit. It was confirmed by pathology that malignant cells were absent in all five lesions. Lu et al. (15) also reported that no characteristics of PTC were found by FNA and CNB at the 3 months and 6 months follow-up in 44 patients with unifocal micro-PTC who underwent MWA. In other studies, the local recurrence rate after MWA treatment was also very low (16–18). Therefore, we concluded that MWA treatment was feasible for the complete destruction of unifocal low-risk micro-PTC.

However, local tumor residue after TA treatment for primary PTC was also reported in several previous studies. Ma et al. (6) retrospectively analyzed postoperative pathology data of 12 patients with primary PTC treated with TA and confirmed residual PTCs in all cases pathologically. Similar results were also reported by Sun et al. (7) and Kim et al. (19). The two distinct conclusions may mainly be due to the selection of patients and ablation methods. In the study of Ma et al., most of the cases (9/12) were PTC (size > 1 cm), whereas only two cases (2/12) were multifocal micro-PTC. In addition, some cases (2/12) had clinically evident cervical lymph node metastasis before MWA. Therefore, these patients had more “high-risk factors” than our patients (20). In our study, one case (1/12) was also diagnosed as PTC (size > 1 cm) and showed residual tumor in postoperative pathology. Although a few studies also reported that TA was feasible in treating patients with solitary T1bN0M0 PTC (21, 22), the need to protect surrounding thyroid tissue made it more difficult to completely eliminate local lesions for PTC (size>1 cm) than for micro-PTC (7). The application of MWA in the treatment of PTC (size>1 cm) is still limited and controversial. There were three cases of multifocal micro-PTC in our study, and in two of these cases (2/12), complete tumor elimination was achieved. Regarding multifocal micro-PTC, the main challenges were the potential occult micro-PTC and the tendency of recurrence. Multifocality is also a risk factor of cervical lymph node metastasis (20). The two most recent guidelines did not recommend using TA for treating multifocal micro-PTC (13, 23). Interestingly, the two cases with multifocal micro-PTC that were successfully treated both had one lesion on each lobe. Another case had more than one tumor lesion on one lobe. Sugitani et al. (24) concluded that multiplicity is not associated with tumor enlargement. Whether MWA is feasible for multifocal micro-PTC having one lesion on each lobe is still controversial and needs further research. Regarding the ablation methods, it remains controversial whether MWA is superior to RFA. In hepatic tumors, the potential to completely destroy the tumor tissues may be higher for MWA than for RFA (25). MWA is thought to be less affected by the heat-sink effect, which may be related to local recurrence after RFA (26). However, some studies also reported that RFA and MWA showed comparable results in terms of volume reduction in the treatment of thyroid nodules (27).

In our study, cervical lymph node metastasis was one of the main reasons for further surgical treatment. Moreover, seven cases (7/12) were confirmed to have cervical lymph node metastases by postoperative pathology. The high lymph node metastasis rate may mainly be due to our patients selection criteria. In previous studies, the rate of lymph node metastasis was very low in low-risk micro-PTC treated by TA, ranging from 0.08%–0.4% (8, 14, 16). Li et al. (18) also showed that there was no statistically significant difference in the rate of lymph node metastasis between MWA and surgery. Therefore, MWA is still feasible for the treatment of low-risk micro-PTC, although long-term US monitoring is recommended to detect potential lymph node metastasis. In addition, three cases (3/12) sought for further surgical treatment because of their uncertainty about the efficacy of MWA, and they were all found to be completely disease free after the surgery. Therefore, these patients need more humanistic care to relieve their anxiety. On the other hand, sonologists need to distinguish the ultrasonographic features of thyroid nodules after MWA treatment because they may present suspicious features such as marked hypoechogenicity, heterogeneity, irregular margins, and hardness as measured by elastography, which are misleading and result in anxiety among patients (28).

Regarding the safety of MWA, no complications were noted after MWA treatment in our study. Ablation lesions were found to adhere to the anterior cervical muscle group in three cases, but it did not significantly increase the difficulty of the surgery. The rates of hoarseness were 2.7% and 4.2%, respectively, in the studies by Teng et al. (29) and Li et al. (18). The complications decreased significantly after MWA treatment compared with surgery (18). Therefore, we conclude that MWA is a safe tool for the treatment of micro-PTC.

This study has some limitations. This was merely a cases series study, and more high-quality medical evidence from studies such as prospective randomized controlled trials is needed to prove the efficacy and safety of MWA. In addition, only 12 patients were enrolled in this study; thus, a larger number of cases is also needed in future studies.

## Conclusion

This study demonstrated that MWA could achieve complete local tumor elimination for primary unifocal low-risk micro-PTC (T1aN0M0). After MWA, long-term US monitoring was necessary. It was recommended to undergo further surgery if there was suspected recurrence or lymph node metastasis in the follow-up visit. More humanistic care and professional post-ablation ultrasonography interpretation are needed for patients undergoing MWA. The safety of MWA was also confirmed by postoperative pathology. However, the application of MWA on PTC (size > 1 cm) and multifocal micro-PTC remains controversial.

## Data Availability Statement

The original contributions presented in the study are included in the article/supplementary material. Further inquiries can be directed to the corresponding authors.

## Ethics Statement

The studies involving human participants were reviewed and approved by Ruijin Hospital, Shanghai Jiao Tong University School of Medicine. Written informed consent for participation was not required for this study in accordance with the national legislation and the institutional requirements.

## Author Contributions

MD reviewed the literature and contributed to data analysis and manuscript drafting; G-SW and J-HG were responsible for data collection and the design of the study; D-JS and RZ contributed to conception of the study. R-LX contributed to funding acquisition. JF, S-RW, and H-CW were responsible for the revision of the manuscript, considering and providing important intellectual content; All authors contributed to the article and approved the submitted version.

## Funding

This work was supported by the Project of Shanghai Municipal Health Commission (Grant No. 20214Y0223) and Medical-Engineering Cross Foundation of Shanghai Jiao Tong University (Grant No. YG2019ZDA17).

## Acknowledgments

We thank Yahui Kong for helping with data collection.

## Conflict of Interest

The authors declare that the research was conducted in the absence of any commercial or financial relationships that could be construed as a potential conflict of interest.

## Publisher’s Note

All claims expressed in this article are solely those of the authors and do not necessarily represent those of their affiliated organizations, or those of the publisher, the editors and the reviewers. Any product that may be evaluated in this article, or claim that may be made by its manufacturer, is not guaranteed or endorsed by the publisher.
